# Radiomics Signature: A potential biomarker for the prediction of survival in Advanced Hepatocellular Carcinoma

**DOI:** 10.7150/ijms.55510

**Published:** 2021-03-30

**Authors:** Lingli Li, Xuefeng Kan, Yongjun Zhao, Bo Liang, Tianhe Ye, Lian Yang, Chuansheng Zheng

**Affiliations:** 1 Department of Radiology, Union Hospital, Tongji Medical College, Huazhong University of Science and Technology, Wuhan 430022, China.; 2 Hubei Key Laboratory of Molecular Imaging, Union Hospital, Tongji Medical College, Huazhong University of Science and Technology, Wuhan 430022, China.; 3 Wuhan Zhikai Technology, Wuhan 430074, China.

**Keywords:** radiomics, nomogram, hepatocellular carcinoma, survival

## Abstract

**Objectives:** To develop and validate radiomics nomograms for the pretreatment predictions of overall survival (OS) and time to progression (TTP) in the patients with advanced hepatocellular carcinoma (HCC) treated with apatinib plus transarterial chemoembolization (TACE), and to assess the incremental value of the clinical-radiomics nomograms for estimating individual OS and TTP.

**Methods:** A total of 60 patients with advanced HCC (BCLC stage C) treated with apatinib plus TACE were divided into a training set (n=48) and a validation set (n=12). The predictors identified from the clinical variables and the radiomics signature constructed from the computed tomography images, such as ɑ-fetoprotein level (AFP), formfactor, the grey level co-occurrence matrix, the gray level size zone matrix, and the gray level run-length matrix, were used to build the clinical-radiomics nomograms and the radiomics nomograms for the prediction of OS and TTP.

**Results:** Apatinib plus TACE benefited the patients with advanced HCC, with a 579-day median OS and a 270-day median TTP. The nomograms were built with the radiomics signature and AFP, and achieved favorable prediction efficacy with acceptable calibration curves. Decision curve analyses demonstrated that the clinical-radiomics nomograms outperformed the radiomics nomograms for the predictions of OS and TTP.

**Conclusions:** Apatinib plus TACE may improve OS and prolonged TTP in the patients with advanced HCC. The clinical-radiomics nomograms, a noninvasive pretreatment prediction tool that incorporate radiomics signature and AFP, demonstrated good prediction accuracy for OS and TTP in these patients. These results indicate that the clinical-radiomics nomograms may provide novel insight for precise personalized medicine approaches in the patients with advanced HCC.

## Introduction

Hepatocellular carcinoma (HCC) is a complex disease most commonly related to chronic liver disease. Epidemiological studies of HCC have revealed that it is the fifth most common cancer and the second most frequent cause of cancer-related death 1, with a growing incidence worldwide 2. HCC also represents about 90% of primary liver cancers 2, with a male to female ratio of approximately 2-2.5:1 3. The greatest HCC burden is in the developing world 4. Approximately 25-70% of patients with HCC present with advanced disease at diagnosis, which is regarded as incurable 2, 4. Treatment selection and survival prediction are critical steps in the management of advanced HCC (BCLC stage C).

Targeted drug therapy is the most common treatment for advanced HCC. Sorafenib, which inhibits vascular endothelial growth factor receptors, is regarded as the standard first-line systemic therapy for advanced HCC 2. However, the incidence of drug-related adverse events is high, and these adverse events may reduce adherence to sorafenib and thus have a negative effect on patient prognosis 5.

In addition to its association with adverse events, the high cost of sorafenib also limits its long-term use in the treatment of advanced HCC patients. For these reasons, new targeted drugs for advanced HCC are currently being developed and tested. For instance, apatinib is a novel and highly selective inhibitor of VEGFR2 tyrosine kinase, with a binding affinity 10 times greater than that of sorafenib 6. Apatinib is currently available in mainland China. A series of studies 7-9 found that apatinib has encouraging antitumor properties and is toxicologically tolerable in several malignant tumor cases. Furthermore, another study 10 found that apatinib has similar antitumorigenic and antiangiogenic efficacy to sorafenib in HCC with less toxicity *in vitro* and *in vivo*. These findings provide preclinical evidence supporting the potential application of apatinib to the treatment of HCC. What's more, apatinib has potential survival benefits for patients with advance HCC, as demonstrated by a phase II randomized, open-label trial 11.

In recent years, the combined use of transarterial chemoembolization (TACE) with anti-angiogenic agents in advanced HCC patients has attracted much interest 12-16. Our previous study 17 also showed the median time to progression (TTP) and overall survival (OS) in the TACE-apatinib group was also significantly greater than that of the TACE-alone group after the propensity score matching analysis, which agreed with those of a series of previous studies 12-14. Apatinib plus TACE had certain survival benefits for advanced HCC in patients who experienced progression following TACE, revealing a potentially promising strategy for the treatment of advanced HCC 12. Given this, determining biomarkers predictive of the efficacy of apatinib plus TACE is needed.

Radiomics is an emerging field in which high-dimensional features are mathematically extracted from medical images. This extraction results in the conversion of images into mineable data and the subsequent analysis of these data for support of medical decision-making 18. Such prognostic prediction models may be built from noninvasively extracted radiomics features of tumors and relevant clinical indicators. Recently, radiomics analyses of HCC using computed tomography (CT) and magnetic resonance images have been shown to have high prediction accuracy 19-21, such as Kim et al. 20 found that gadoxetic acid-enhanced magnetic resonance imaging radiomics could be used for the prediction of postoperative early and late recurrence of single HCC. These radiomic analysis of contrast-enhanced CT predictors have mainly based on advanced HCC patients who were treated with sorafenib 21 or microvascular invasion and outcome in hepatocellular carcinoma 19. However, the use of texture analysis as a therapeutic decision-making biomarker or predictive biomarker for treatment efficacy in cases of advanced HCC treated with apatinib plus TACE has never been investigated.

The aim of present study was to develop and validate radiomics nomograms for the pretreatment predictions of OS and TTP in patients with advanced HCC who were treated with apatinib plus TACE, and to assess the incremental value of such clinical-radiomics nomograms in individual OS and TTP estimation.

## Materials and methods

### Subjects

Ethical approval by the institutional review board was obtained for this retrospective analysis and the requirement for informed consent was waived. This study was conducted in accordance with the Declaration of Helsinki.

Between January 2014 and June 2018, a total of 60 consecutive patients with advanced HCC (BCLC stage C) who received apatinib plus TACE were enrolled in the present study. Patient recruitment (Figure S1) and inclusion and exclusion criteria are depicted in the Supplementary Data. A detailed version of the apatinib plus TACE administration protocol used here is also given in the Supplementary Data. Computer-generated random numbers were used to assign a training set to validation set ratio of about 4:1. Recorded demographic characteristics and clinical data are detailed in the Supplementary Data.

### End Points

End points were chosen according to the guidelines of the American Association for the Study of Liver Diseases 22. The primary end point was OS, defined as the time from the first TACE procedure to the patient's death. TTP (the time from the start of the first TACE procedure to the time of tumor progression as defined by modified Response Evaluation Criteria in Solid Tumors 23) was defined as the secondary end point. Patient follow-up is detailed in the Supplementary Data. Patients alive or those without radiologic progression at the end of the follow-up period were removed.

### Median OS and median TTP

Median OS and median TTP of the full cohort (all 60 patients) were calculated using Kaplan-Meier analyses.

### CT image acquisition, region-of-interest segmentation, and radiomics features extraction

**Figure 1:**
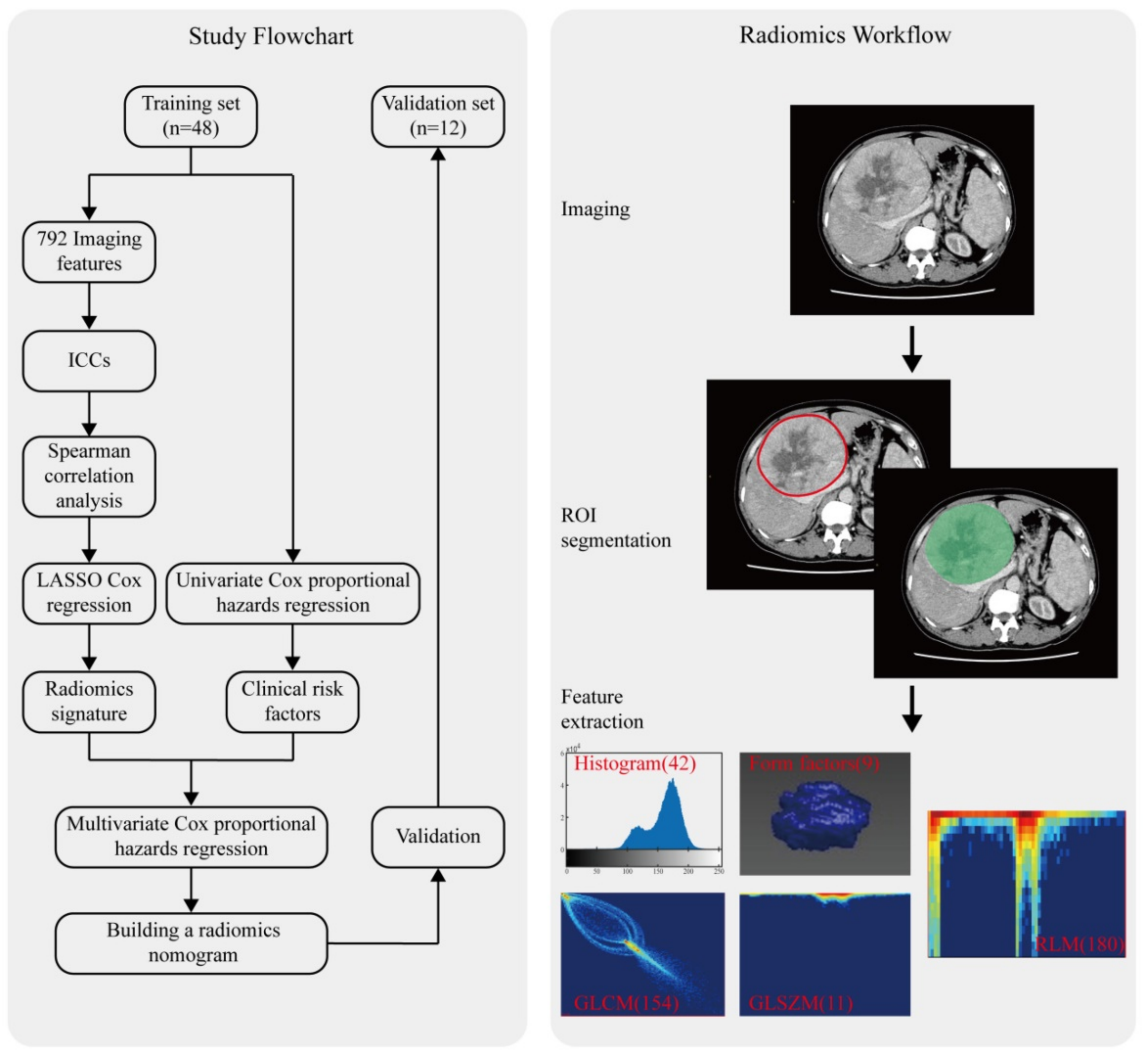
Radiomics workflow and study flowchart.

The radiomics workflow used in the present study is depicted in Figure 1. A detailed CT protocol is also given in the Supplementary Data. Tumor regions of interest (ROIs) were hand-drawn on both the late arterial phase and the portal venous phase of pretreatment contrast-enhanced CT images on each slice by two radiologists with more than 10 years of experience. Itk-SNAP software (www.itk-snap.org) was used for manual segmentation. Radiomics features for each patient were extracted using Artificial Intelligence Kit version 1.0.3 (GE Healthcare, Boston USA). In total, 396 texture parameters from the late arterial phase and 396 texture parameters from the portal venous phase were extracted from a single CT image of each patient. The radiomics features were classified into six categories (Supplementary Table S1): histogram, the grey level co-occurrence matrix (GLCM), the gray level size zone matrix (GLSZM), the gray level run-length matrix (RLM), and shape- and size-based features. More detailed information about the radiomics features can be found in the Supplementary Data.

Intra- and inter-reader agreement for the texture parameters were assessed using intraclass correlation coefficients (ICCs) for each pair of variables in the whole sample. To assess intra-observer reproducibility, reader 1 then repeated the same manual procedure one week later. An ICC greater than 0.7 was considered good feature extraction agreement 21, 24. Correlations between texture parameters were assessed using Spearman correlations, with correlation coefficients more than 0.9 considered significant.

### Feature selection and radiomics signature construction

The least absolute shrinkage and selection operator (LASSO) Cox regression algorithm was used to analyze all high-dimensional data. A LASSO Cox regression was applied to select OS- and TTP-related features with nonzero coefficients from a subset of features out of the 792 texture parameters in the training set after performing the spearman correlation analysis. Penalty parameter tuning was conducted with 10-fold cross-validation. Additional details on feature processing and selection can be found in our prior work 25. A radiomics score (Rad-score) was generated using a linear combination of selected features that were weighted by their respective LASSO Cox regression coefficients. The potential survival predictors among clinical variables, such as AFP, total bilirubin, and Child-Pugh class, were identified using a univariate Cox proportional hazards regression analysis approach.

### Construction of the radiomics nomogram

The radiomics signature and clinical predictors were tested via a multivariate Cox proportional hazards regression model to predict OS and TTP in the training set. To provide the clinician with a quantitative tool to predict individual probability of OS and TTP, we built the clinical-radiomics nomograms and radiomics nomograms on the basis of multivariate Cox proportional hazards regression analysis with the training set.

### Assessment of nomogram performance

Nomogram calibration was assessed with a calibration curve. Harrell's C-index was calculated, which was applied to quantify the discrimination performance of the radiomics nomogram. The clinical-radiomics nomograms and radiomics nomograms were subjected to bootstrapping validation (1,000 bootstrap resamples) to calculate a relatively corrected C-index 26.

### Internal validation of the radiomics nomogram

The performance of the internally-validated radiomics nomogram was assessed with the validation set. Using the formula constructed in the training set, a Rad-score was calculated for each patient in the validation set. Harrell's C-index and the relatively corrected C-index were calculated, and the resultant calibration curves were obtained.

### Clinical utility of the radiomics nomogram

Decision curve analysis (DCA) was conducted to determine the clinical usefulness of the clinical-radiomics nomograms by calculating the net benefits for a range of threshold probabilities on the full cohort with 5-fold cross-validation 27.

### Statistical analyses

Statistical analyses were conducted with R software (version 3.5.3, http://www.Rproject.org) and Python3.7. Package details are available in the Data Supplement. A two-sided P <0.05 was considered significant.

## Results

### Patient characteristics and univariate analyses of training set outcomes

The study flowchart is presented in Figure 1. The main clinical characteristics of patients in the training and validation sets are shown in Table 1. The median OS in the whole cohort was 579 days (range, 90-1975) and the median TTP was 270 days (range, 30-1006). Demographic and pretreatment clinical characteristics did not significantly differ between the training and validation sets, except for a significantly lower pretreatment serum albumin level and tumor size in the validation set (P = 0.017, P=0.018). The median OS was 480 days (range, 90-1975) in the training set and 570 days (range, 180-1468) in the validation set (P = 0.496). The median TTP in the training and validation sets were 240 (range, 30-1006) and 275 (range, 60-458) days, respectively (P = 0.28).

Supplementary Table S2 contains the results of a univariate cox proportional hazards regression analyses of pretreatment clinical characteristics, for predicting OS and TTP in the training set. Among the pretreatment demographic and clinical parameters, ɑ-fetoprotein (AFP) level (hazard ratio [HR], 1.000021; 95% confidence interval [CI]: 1.000007, 1.000035; P<0.005) was significantly associated with OS. AFP level (HR, 1.00002; 95% CI: 1.00001, 1.00003; P<0.005) was also significantly associated with TTP.

### Feature selection and radiomics signature construction

The inter- (ICC, range 0.70-0.98) and intra-observer (ICC, 0.70-0.96) ratings were high, indicating favorable intra- and inter-observer feature extraction reproducibility. Given this, all outcomes were based on the measurements obtained by the first radiologist. All features with spearman correlation coefficients greater than 0.9 were removed. A total of 57 radiomics features for each patient were reserved from the late arterial (29 features) and portal venous phases (28 features).

The 57 radiomics features of advanced HCC from the late arterial and venous phases of pretreatment contrast-enhanced CT images were reduced to 8 potential predictors of OS on the basis of 48 patients in the training set (Supplementary Table S3A). These features are of nonzero coefficients in the LASSO logistic regression algorithm. Similarly, 12 potential predictors of TTP were reserved (Supplementary Table S3B). These radiomics features were included in the Rad-score calculation formula (Supplementary Data).

### Construction of the radiomics nomogram

**Figure 2:**
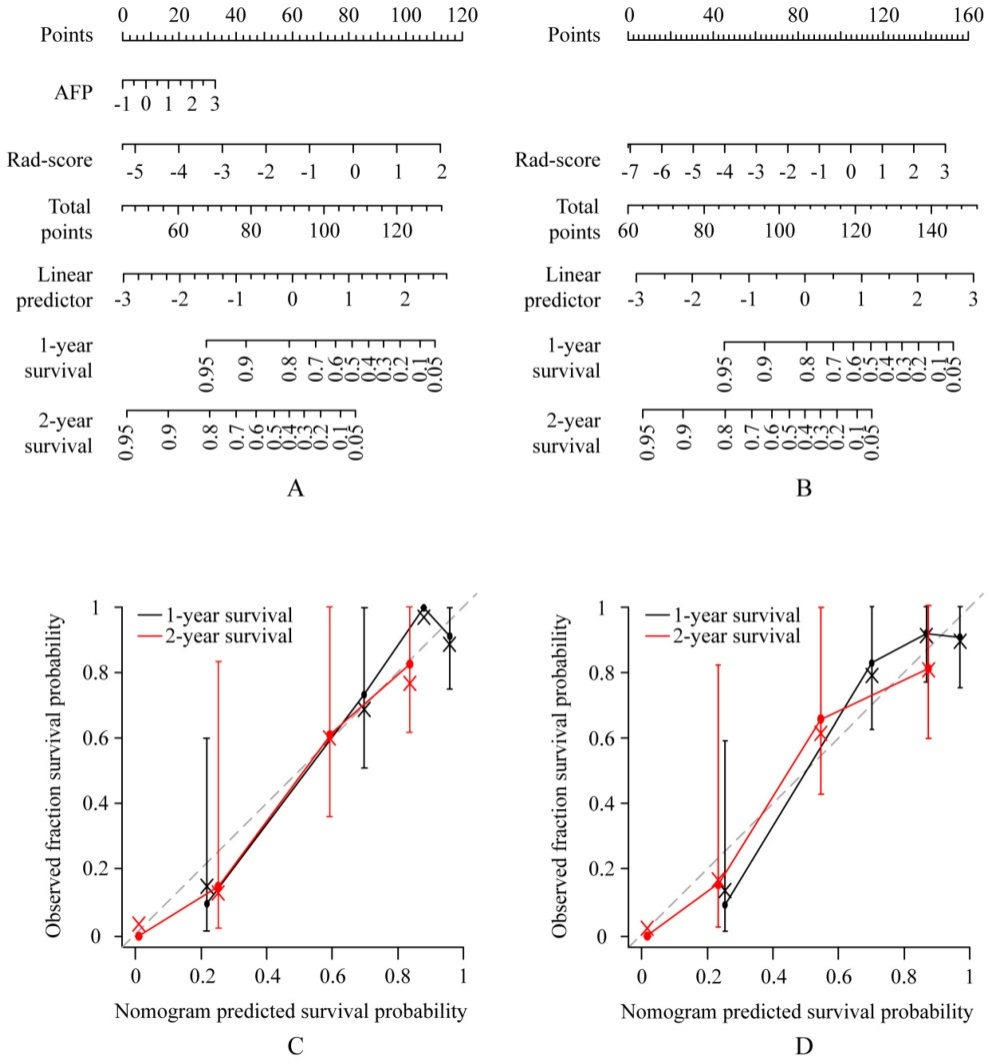
** Use of the constructed clinical-radiomics nomogram and radiomics nomogram to predict overall survival (OS) in patients with advanced HCC, along with the assessment of the model calibration.** Clinical-radiomics nomogram (A) and radiomics nomogram (B). Locate the patient's Rad-score on the Rad-score axis. Draw a line straight upward to the points' axis to determine how many points toward the probability of OS the patient receives for his or her Rad-score. Repeat the process for each variable. Sum the points achieved for each of the risk factors. Locate the final sum on the Total Point axis. Draw a line straight down to find the patient's probability of OS. Calibration curves for the clinical-radiomics nomogram (C) and radiomics nomogram (D) show the calibration of each model in terms of the agreement between the predicted and the observed 1-and 2-year outcomes. Nomogram predicted OS is plotted on the x-axis; the observed fraction OS is plotted on the y-axis. Diagonal dotted line = a perfect prediction by an ideal model, in which the predicted outcome perfectly corresponds to the actual outcome. Solid line = performance of the nomogram, a closer lining of which with the diagonal dotted line represents a better prediction.

**Figure 3:**
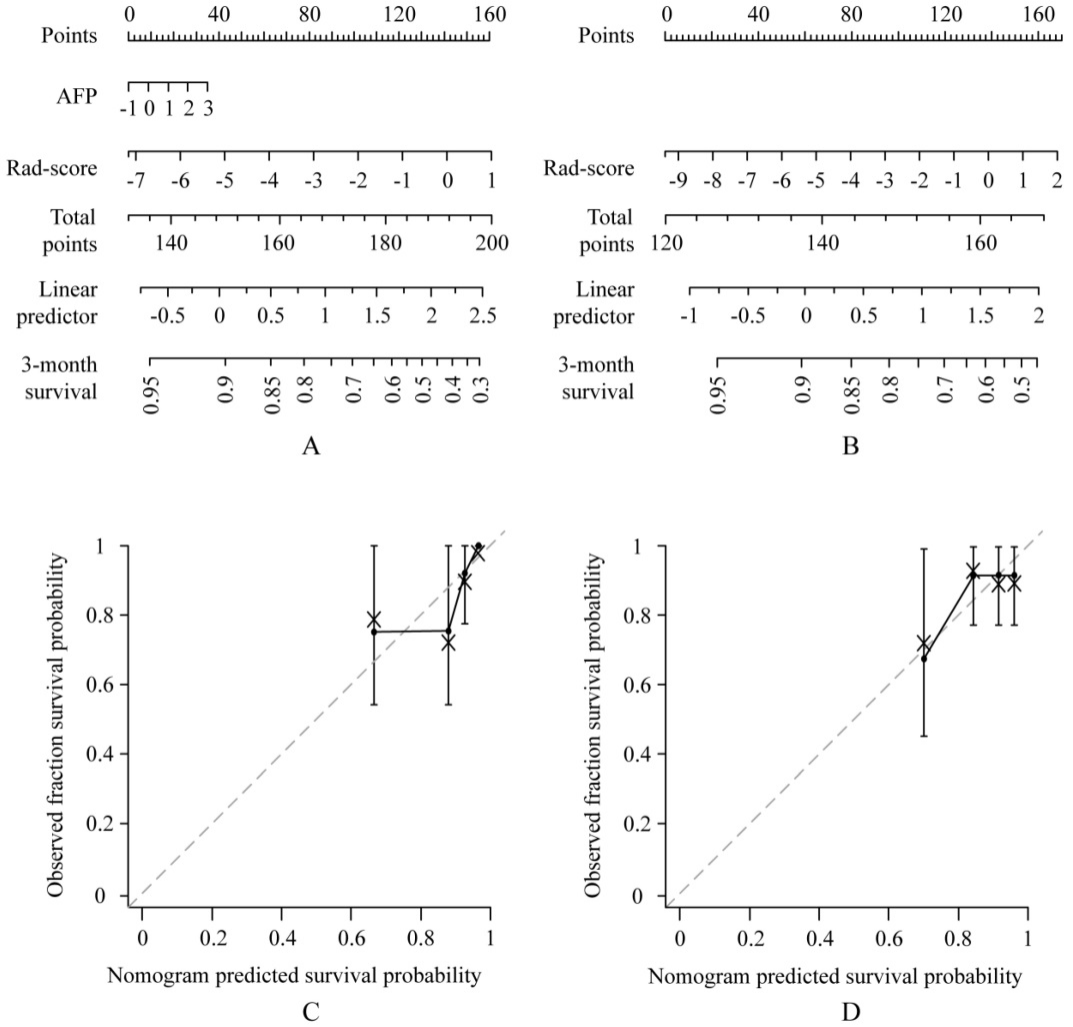
** Use of the constructed clinical-radiomics nomogram and radiomics nomogram to predict time to progression (TTP) in patients with advanced HCC, along with the assessment of the model calibration.** Clinical-radiomics nomogram (A) and radiomics nomogram (B). Locate the patient's Rad-score on the Rad-score axis. Draw a line straight upward to the points' axis to determine how many points toward the probability of TTP the patient receives for his or her Rad-score. Repeat the process for each variable. Sum the points achieved for each of the risk factors. Locate the final sum on the Total Point axis. Draw a line straight down to find the patient's probability of TTP. Calibration curves for the clinical-radiomics nomogram (C) and radiomics nomogram (D) show the calibration of each model in terms of the agreement between the predicted and the observed 3-month outcomes. Nomogram predicted TTP is plotted on the x-axis; the observed fraction TTP is plotted on the y-axis. Diagonal dotted line = a perfect prediction by an ideal model, in which the predicted outcome perfectly corresponds to the actual outcome. Solid line = performance of the nomogram, a closer lining of which with the diagonal dotted line represents a better prediction.

A multivariate Cox proportional hazards regression analysis identified the radiomics signature and AFP as independent predictors (Table 2, Table 3, Supplementary Table S4A-Table S4B). The models that incorporated these independent predictors were developed and presented as the nomograms (Figure 2A-B, Figure 3A-B).

### Apparent performance of the radiomics nomogram in the training set and validation in the validation set

The calibration curves of the radiomics nomograms and the clinical-radiomics nomograms for OS and TTP demonstrated good agreement between prediction and observation in the training set (Figure 2C-D, Figure 3C-D). The C-index for the clinical-radiomics nomogram-based prediction of OS and TTP for the training set were 0.833 (95% CI, 0.793 to 0.873) and 0.739 (95% CI, 0.692 to 0.786), then confirmed to be 0.792 (95% CI, 0.715 to 0.870) and 0.701 (95% CI, 0.635 to 0.767) via bootstrapping validation in the validation set, respectively. While the C-index for the radiomics nomogram-based prediction of OS and TTP for the training set were 0.828 (95% CI, 0.787 to 0.869) and 0.732 (95% CI, 0.685 to 0.779), then confirmed to be 0.745 (95% CI, 0.695 to 0.795) and 0.586 (95% CI, 0.543 to 0.628) via bootstrapping validation in the validation set, respectively.

### Clinical utility of the radiomics nomogram

**Figure 4:**
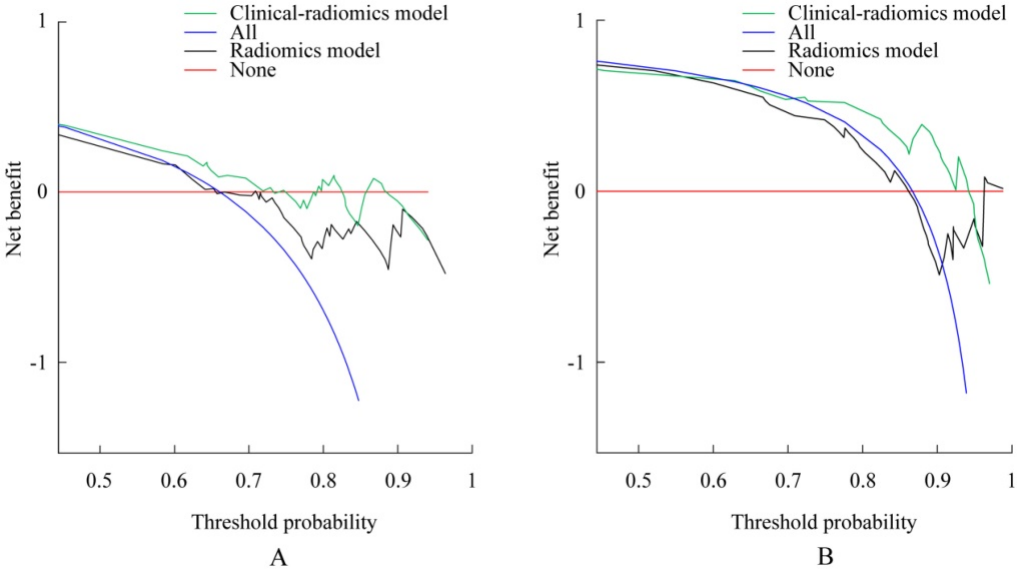
** Decision curve analysis for each model.** Decision curve for the models of prediction overall survival (OS) with 1-year survival probability. (B) Decision curve for the models of prediction time to progression (TTP) with 3-month survival probability. The y-axis measures the net benefit. The net benefit was calculated by summing the benefits (true-positive results) and subtracting the harms (false-positive results), weighting the latter by a factor related to the relative harm of an undetected cancer compared with the harm of unnecessary treatment. The clinical-radiomics model had the highest net benefit compared with radiomics model and simple strategies such as follow-up of all patients (blue line) or no patients (red line) across the full range of threshold probabilities at which a patient would choose to undergo imaging follow-up.

A decision curve analysis revealed that the clinical-radiomics nomograms developed here had higher overall net benefit than the radiomics nomograms across most of the range of reasonable threshold probabilities for both OS and TPP (Figure 4).

## Discussion

This study presents a retrospective analysis to develop and validate radiomics nomograms for the pretreatment predictions of OS and TTP in patients with advanced HCC who were treated with apatinib plus TACE, and to assess the incremental value of such clinical-radiomics nomograms in individual OS and TTP estimation, which is an important complement to these previous studies 19-21. Collectively, the results of present study suggest that apatinib plus TACE may improve OS and prolong TTP in patients with advanced HCC. These results agree with those of a series of previous studies 12-14, 17. When compared to a median survival time of 6-8 months or 25% at one year in patients with advanced HCC (BCLC stage C), as described by the EASL clinical practice guidelines 2, the results of the present study indicate that apatinib plus TACE may improve OS in patients with advanced HCC. Similarly, when compared to a median TTP of 2.8 months in sorafenib-treated patients 28, the results of the present study reveal that apatinib plus TACE may prolong TTP in patients with advanced HCC. Though the present study found that apatinib plus TACE may be a promising strategy for the treatment of advanced HCC, the present study's sample size was relatively small. Future investigations should utilize a larger sample size and multicenter validation approaches to establish stronger evidence for the broad clinical application of these treatments.

A multi-feature-based radiomics signature was identified in present study to be an independent biomarker for OS and TTP in patients with advanced HCC treated with apatinib plus TACE. The combined use of a radiomics signature and AFP in the clinical-radiomics nomograms performed better than the radiomics nomograms alone. Prior studies 21, 29, 30 have also shown that several clinical parameters, including macroscopic vascular invasion, AFP, serum albumin levels, and Child-Pugh class, are associated with clinical outcomes in patients with HCC. Our results show that AFP is associated with OS and TTP, a finding which is consistent with prior work 21, 29, 30. Macroscopic vascular invasion, Child-Pugh class, and serum albumin levels have been previously significantly associated with OS 21, 29, 31, though they were not in the present study.

The radiomics features were derived from CT images both in the late arterial and portal venous phases of the patients. The radiomics signature identified from these features included those both in the late arterial and portal venous phases when building clinical-radiomics and radiomics nomograms for OS, as well as the radiomics nomogram for TTP. However, the radiomics signature only included radiomic features in the late arterial phase for building clinical-radiomics nomogram for TTP. Tumor heterogeneity is closely related to tumor prognosis, most notably in HCC lesions 32. Heterogeneous arterial phase enhancement seems to be predictive of high tumor grade and recurrence after treatment in HCC. However, advanced HCC often appears to be hypovascular, with decreased arterial flow 33. This may explain the prognostic significance of radiomics signature, which reflect physiological heterogeneity and can be estimated at the portal venous phase. Our results collectively demonstrate that increased pretreatment tumor inhomogeneity is associated with poor clinical outcomes in patients with advanced HCC. These findings are consistent with those of previous HCC studies 19, 21.

As demonstrated in present study, the radiomics signature may predict survival outcomes, supporting the conclusion that radiomics signature can obtain intratumoural heterogeneity in a noninvasive way that is relevant to patient prognosis. Furthermore, the radiomics signature and clinical-radiomics nomogram used here was demonstrated to accurately estimate OS and TTP. When compared with long-term OS outcomes, TTP was defined as a secondary end point associated with less extended follow-up and thus more effective therapeutic adjustment 23. Thus, the present study proposes an efficient and noninvasive pretreatment prediction tool that enables earlier development of personalized treatment approaches.

The radiomics nomogram, as a statistical model, accounts for multiple risk factors by assigning a total number of points to each patient. A series of studies 26, 34-36 have shown that the radiomics nomogram can effectively and comprehensively predict the post-operative outcomes of individual patients. Furthermore, pretreatment portal venous phase-derived tumor entropy may be a predictor of survival in patients with advanced HCC treated with sorafenib 21. As the first study of a radiomics signature for prediction of OS and TTP in patients with advanced HCC treated with apatinib plus TACE, the present study demonstrated that the use of a clinical-radiomics nomogram achieved superior prognostic performance than a radiomics nomogram alone, with a higher C-index and better calibration. The utility of this proposed nomogram was confirmed in a validation set. A decision curve analysis also revealed that a clinical-radiomics nomogram was superior to a radiomics nomogram across most of the range of reasonable threshold probabilities tested here.

Limitations of present study include its retrospective design, a limited sample size, lack of a control group, and lack of an external model validation. A large-scale independent prospective multicenter validation cohort is thus needed to acquire high-level evidence for broader clinical application. In addition, no gene-expression signature has been incorporated into the nomogram used here. Tumor gene expression patterns can provide insights into patient prognosis 37. However, intratumor heterogeneity has also led to an underestimation of the tumor genomics landscape, as revealed by single tumor-biopsy samples. This may contribute to treatment failure and drug resistance 38.

## Conclusions

Apatinib plus TACE may improve OS and prolong TTP in the patients with advanced HCC. The clinical-radiomics nomograms, a noninvasive pretreatment prediction tool that incorporate radiomics signature and AFP, demonstrated good prediction accuracy for OS and TTP in these patients. These results indicate that the clinical-radiomics nomograms may provide novel insight for precise personalized medicine approaches in the patients with advanced HCC.

## Supplementary Material

Supplementary figures and tables.

## Figures and Tables

**Table 1:** Main baseline demographic and clinical characteristics of patients in the training set and validation set

Characteristic	Training set (n = 48)	Validation set (n = 12)	*P*-value
**Sex**			0.998
Male	41 (85)	10 (83)	
Female	7 (15)	2 (17)	
Median age (y)*	49 (31-66)	49 (36-59)	0.318
Median BMI (kg/m^2^)*	21.9 (18.3-28.7)	23.5 (18.4-27.9)	0.134
**Cause of disease**			0.997
Chronic hepatitis B only	43 (90)	11 (92)	
Unknown	5 (10)	1 (8)	
**Child-Pugh class**			
A	41 (85)	9 (75)	0.861
B	7 (15)	3 (25)	
**ECOG performance status**			0.862
0	7 (15)	1 (8)	
1	36 (75)	8 (67)	
2	5 (10)	3 (25)	
**HCC type**			0.717
Nodular	23 (48)	8 (67)	
massive	25 (52)	4 (33)	
Median tumor size (mm)	79 (32-160)	60 (35-121)	0.018^#^
Macroscopic vascular invasion	28 (58)	6 (50)	0.965
Extrahepatic spread	30 (63)	7 (58)	0.995
Biochemical analysis*			
Median albumin level (g/dL)	37 (25-45)	34 (31-39)	0.017^#^
Median total bilirubin level (mg/dL)	16 (6-41)	19 (10-101)	0.216
Median ɑ-fetoprotein level (ng/mL)	760 (1-84500)	336 (3-72814)	0.419
Median survival end points (d)*			
Overall survival	480 (90-1975)	570 (180-1468)	0.496
Time to progression	240 (30-1006)	275 (60-458)	0.280

Note: Except where indicated, data are numbers of patients, with percentages in parentheses. BMI: body mass index, ECOG: Eastern Cooperative Oncology Group, HCC: hepatocellular carcinoma; *Numbers in parentheses are ranges; #Statistically significant.

**Table 2:** Multivariate Cox proportional hazards regression analyses of the advanced HCC radiomics signature and clinical features for predicting overall survival in the training set

Radiomics signature and clinical feature	Hazard Ratio*	*P*-value
ɑ-fetoprotein level	1.67 (1.11-2.53)	0.01^#^
InverseDifferenceMoment_AllDirection_offset4_SD (art)	0.72 (0.47-1.09)	0.12
Compactness1 (art)	0.65 (0.43-0.97)	0.04^#^
ClusterShade_AllDirection_offset1 (por)	2.53 (1.58-4.48)	<0.005^#^
ZonePercentage (por)	1.73 (1.20-2.48)	<0.005^#^

Note.-HCC: hepatocellular carcinoma, art: late arterial phase, por: portal venous phase; *Numbers in parentheses are 95% confidence intervals; #Statistically significant.

**Table 3:** Multivariate Cox proportional hazards regression analyses of the advanced HCC radiomics signature and clinical features for predicting time to progression in the training set

Radiomics signature and clinical feature	Hazard Ratio*	*P*-value
ɑ-fetoprotein level	1.54 (1.05-2.27)	0.03^#^
ShortRunEmphasis_AllDirection_offset1_SD (art)	2.33 (1.20-4.53)	0.01^#^
Compactness1 (art)	0.66 (0.46-0.93)	0.02^#^
ShortRunHighGreyLevelEmphasis_AllDirection_offset1_SD (art)	0.53 (0.30-0.92)	0.03^#^
LargeAreaEmphasis (art)	1.60 (1.13-2.27)	0.01^#^
LowGreyLevelRunEmphasis_AllDirection_offset4_SD (art)	0.73 (0.48-1.11)	0.13

Note.-HCC: hepatocellular carcinoma, art: late arterial phase, por: portal venous phase; *Numbers in parentheses are 95% confidence intervals; #Statistically significant.
