# Supplementary material for: Radiomics Signature: A potential biomarker for the prediction of survival in Advanced Hepatocellular Carcinoma
Source: Int J Med Sci. 2021 Mar 30;18(11):2276–84. doi: 10.7150/ijms.55510 (PMC8100633; doi:10.7150/ijms.55510)

I. Supplementary Data

1. Study Population

1.1 Recorded demographics and clinical data

Recorded demographics and clinical data included patients age, sex, body mass index (BMI, kg/m^2), Eastern Cooperative Oncology Group (ECOG) performance status score, cause of hepatic disease, Child-Pugh class (A or B), nodular or infiltrative type of HCC, size of the HCC lesion chosen for CT texture analysis, presence or absence of proximal macroscopic vascular invasion (MVI, portal vein and/or right or left branches), presence or absence of extrahepatic spread, serum levels of alpha-fetoprotein, albumin, and total bilirubin, start date of apatinib plus TACE treatment, and date of pretreatment CT examination.

1.2 Inclusion and exclusion criteria

The inclusion criteria for our study were as follows: they were receiving apatinib plus TACE for advanced HCC (Barcelona Clinic Liver Cancer stage C) [1] and had undergone baseline contrast-enhanced CT within 2 months before treatment initiation. The exclusion criteria for our study were as follows: infiltrative HCC without any accurately delineable lesion; died during the first 15 days after apatinib plus TACE treatment initiation; and were without a regular documented follow-up during the 1st year after apatinib plus TACE initiation or until disease progression.

Reference:1. Llovet, J.M., C. Bru, and J. Bruix, Prognosis of hepatocellular carcinoma: the BCLC staging classification. Semin Liver Dis, 1999. 19(3): p. 329-38.

2. apatinib plus TACE protocol

2.1 TACE procedure

TACE was performed by an interventional radiologist with at least 8 years of TACE experience. Initially, a tip of 3-French microcatheter (Progreat, Terumo, Tokyo, Japan) or 5-French catheter (Cook, Bloomington, Indiana, USA) was advanced into the tumor-feeding arteries. Then, 10-20 mL lipiodol (Lipiodol Ultrafluido, Guerbet, France) was mixed with 20-40 mg doxorubicin hydrochloride (Hisun Pharmaceutical Co. LTD, Zhejiang, China) to create an emulsion. Depending on the tumor size and liver function, 5-20 mL of the emulsion was injected into the tumor feeding arteries through the 5-French catheter or 3-French microcatheter. Lastly, gelatin sponge particles (300-700 um, Cook, USA) was used for embolization until the stasis of arteries flow was appeared. For patients with arterioportal shunt, polyvinyl alcohol particles (300-1000 um, Cook, USA) was used for occluding the shunt before infusion the emulsion of lipiodol and doxorubicin.

The TACE was repeated at least 40 days after the first treatment in patients with survival lesions according to the mRECIST [1], TACE was not repeated until liver failure or tumor progression of the target lesions was observed.

Reference :1. Lencioni, R. and J.M. Llovet, Modified RECIST (mRECIST) assessment for hepatocellular carcinoma. Semin Liver Dis, 2010. 30(1): p. 52-60.

2.2 Apatinib administration

Upon agreeing to participate in the study, apatinib was orally taken at 3-5 days after each TACE procedure, and the initial dose was 500 mg/day. When the patients encountered grade 3-4 drug-related AEs according to the Common Terminology Criteria for Adverse Events version 4.0, the drug dose was adjusted to 250 mg/day or stopped for several days. After the adverse events

were relieved, the patients were recommended to resume daily intake of 500 mg/day apatinib. Treatment continued until patient death, significant disease progression, drug intolerance, or withdrawal of consent from the study.

3. Patient Follow-up

All patients underwent clinical and radiologic follow-up according to institutions protocol. Radiological follow-up consisted of multiphase contrast-enhanced CT scans including at least arterial and portal-venous phases, scheduled every 3 months. Additional CT scan was performed in case of clinical suspicion of disease progression or acute complication. Any tumor progression described during follow-up was reviewed for validation at local regular multidisciplinary HCC meeting.

4. The radiomics procedure

4.1 CT image acquisition and retrieving procedure

All patients underwent contrast-enhanced abdominal CT with a 64-slice spiral CT scanner (Somatom Sensation 64, Siemens Healthineers Ltd., Forcheim, Germany). The CT scan parameters were as follows: 120 kV, CARE Dose 4D, 200 effective mAs, beam collimation of 64 x 0.6 mm, a matrix of 512 x 512, a pitch of 0.8, and a gantry rotation time of 0.5 s. After nonenhanced CT scanning, a dynamic contrast-enhanced CT scan was performed after intravenous administration of 80-100 mL nonionic contrast material (Iopamidol, 370 mg I/mL, Bracco) using power injection at a rate of 3.5 mL/second followed by saline flush (20 mL).Arterial-phase and vein-phase images were obtained at 25 and 60 seconds, respectively. The slice thickness of the reconstructed image was 5.0 mm, and the kernel was B30f. The arterial phase and the portal venous

phase CT images (thickness: 5mm) were retrieved from the picture archiving and communication system (PACS) (Carestream, Canada) for image feature extraction.

4.2 Texture analysis methodology

Texture analysis was applied to the CT images using in-house texture analysis software(Analysis Kit, version 3.1.5.R, GE Healthcare). A region of interest (ROI) was delineated initially around the tumor outline for the 3D ROI area. In total, 396 imaging texture features from the category of histogram, the Grey level co-occurrence matrix (GLCM), the gray level size zone matrix (GLSZM), the gray level Run-length matrix (RLM), and Shape and size based features were finally extracted from one single image **Table S1.**

Table S1:Summary of radiomic features used in this study

Feature classes	No. of features	3 representative features
Histogram	42	FrequencySize, MaxIntensity, MeanValue,…
GLCM	144	ClusterProminence, ClusterShade, Correlation,…
GLSZM	11	SizeZoneVariability, HighIntensityEmphasis, IntensityVariability,…
RLM	180	GreyLevelNonuniformity, HighGreyLevelRunEmphasis, LongRunEmphasis,…
Formfactor	9	Compactness1, Maximum3DDiameter, Sphericity,…
Haralick	10	HaraEntroy, contrast, differenceEntropy,…
Total	396	

GLCM= the Grey level co-occurrence matrix, GLSZM =the gray level size zone matrix, RLM= the gray level Run-length matrix

(1) **The Grey level co-occurrence matrix (GLCM)**

$\mathbf{P}\ (\mathbf{i},\mathbf{j}|\boldsymbol{\theta},\mathbf{d})$ represents the joint probability of certain sets of pixels having certain grey-level values. It calculates how many times a pixel with grey-level **i** occurs jointly with another pixel having a grey value **j**. By varying the displacement vector **d** between each pair of pixels.

The rotation angle of an offset: 0 °,45 °,90 °,135 °and displacement vectors (distance to the neighbor pixel: 1, 2, 3 ...), different co-occurrence distributions from the same image of reference. GLCM of an image is computed using displacement vector d defined by its radius, (distance or count to the next adjacent neighbor preferably is equal to one) and rotational angles.

1) Energy of GLCM

Formula

$$\sum_{i,j} g(i,j)^2$$

*g is a GLCM, Where i,j are the spatial coordinates of g (i,j).

This feature Returns the sum of squared elements in the GLCM. Range = [0 1].Energy is 1 for a constant image. Is high when image has very good homogeneity or when pixels are very similar The Property Energy is also known as uniformity, uniformity of energy, and angular second moment.

In AK Software we have 18 parameters related to the GLCM Energy :

GLCMEnergy_AllDirection_offset1, GLCMEnergy_AllDirection_offset1_SD,

GLCMEnergy_angle0_offset1, GLCMEnergy_angle45_offset1,

GLCMEnergy_angle90_offset1, GLCMEnergy_angle135_offset1,

GLCMEnergy_AllDirection_offset4, GLCMEnergy_angle0_offset4,

GLCMEnergy_angle45_offset4, GLCMEnergy_angle90_offset4,

GLCMEnergy_angle135_offset4, GLCMEnergy_AllDirection_offset4_SD,

GLCMEnergy_AllDirection_offset7, GLCMEnergy_angle0_offset7,

GLCMEnergy_angle45_offset7, GLCMEnergy_angle90_offset7,

GLCMEnergy_angle135_offset7, GLCMEnergy_AllDirection_offset7_SD

2) Entropy of GLCM

Formula

$$-\sum_{i,j} g(i,j) \log_2 g(i,j)$$

Entropy is a measure of randomness of intensity image.

Entropy shows the amount of information of the image that is needed for the image compression. Entropy measures the loss of information or message in a transmitted signal and also measures the image information.

In AK Software we have the 18 parameters related to the GLCM Entropy

GLCMEntropy_AllDirection_offset1, GLCMEntropy_AllDirection_offset1_SD,

GLCMEntropy_angle0_offset1, GLCMEntropy_angle45_offset1,

GLCMEntropy_angle90_offset1, GLCMEntropy_angle135_offset1,

GLCMEntropy_AllDirection_offset4, GLCMEntropy_AllDirection_offset4_SD,

GLCMEntropy_angle0_offset4, GLCMEntropy_angle45_offset4,

GLCMEntropy_angle90_offset4, GLCMEntropy_angle135_offset4,

GLCMEntropy_AllDirection_offset7, GLCMEntropy_AllDirection_offset7_SD,

GLCMEntropy_angle0_offset7, GLCMEntropy_angle45_offset7,

GLCMEntropy_angle90_offset7, GLCMEntropy_angle135_offset7

3) Inertia of GLCM

Formula

$$\sum_{i,j}((i-j)^2 g(i,j))$$

It reflects the clarity of the image and texture groove depth. The contrast is proportional to the texture groove, high values of the groove produces more clarity, in contrast small values of the groove will result in small contrast and fuzzy image.

In AK Software we have the 18 parameters related to the Inertia.

Inertia_AllDirection_offset1, Inertia _AllDirection_offset1_SD,

Inertia _angle0_offset1, Inertia _angle45_offset1,

Inertia _angle90_offset1, Inertia _angle135_offset1,

Inertia _AllDirection_offset4, Inertia _AllDirection_offset4_SD,

Inertia _angle0_offset4, Inertia _angle45_offset4,

Inertia _angle90_offset4, Inertia _angle135_offset4,

Inertia _AllDirection_offset7, Inertia _AllDirection_offset7_SD,

Inertia _angle0_offset7, Inertia _angle45_offset7,

Inertia _angle90_offset7, Inertia _angle135_offset7

4) Correlation

Formula

$$-\sum_{i,j}\frac{(i-\mu)(j-\mu)g(i,j)}{\sigma^2}$$

Image-based Correlation measures the similarity of the grey levels in neighboring pixels, tells how correlated a pixel is to its neighbor over the whole image. Range = [-1 1]. Correlation is 1 or -1 for a perfectly positively or negatively correlated image.

In AK Software we have the 18 parameters related to the Inertia.

Correlation_AllDirection_offset1, Correlation _AllDirection_offset1_SD,

Correlation _angle0_offset1, Correlation _angle45_offset1,

Correlation _angle90_offset1, Correlation _angle135_offset1,

Correlation _AllDirection_offset4, Correlation _AllDirection_offset4_SD,

Correlation _angle0_offset4, Correlation _angle45_offset4,

Correlation _angle90_offset4, Correlation _angle135_offset4,

Correlation _AllDirection_offset7, Correlation _AllDirection_offset7_SD,

Correlation _angle0_offset7, Correlation _angle45_offset7,

Correlation _angle90_offset7, Correlation _angle135_offset7.

5) Inverse Difference Moment

Formula

$$\sum_{i,j} \frac{1}{1+\ (i-j)^{2}} g(i,j)$$

Inverse Difference Moment (IDM) is the local homogeneity. It is high when local gray level is uniform. IDM weight value is the inverse of the Contrast weight.

In AK Software we have the 18 parameters related to the Inertia.

InverseDifferenceMoment_AllDirection_offset1,

InverseDifferenceMoment_AllDirection_offset1_SD

InverseDifferenceMoment_AllDirection_offset4,

InverseDifferenceMoment_AllDirection_offset4_SD

InverseDifferenceMoment_AllDirection_offset7,

InverseDifferenceMoment_AllDirection_offset7_SD

InverseDifferenceMoment_angle0_offset1, InverseDifferenceMoment_angle0_offset4

InverseDifferenceMoment_angle0_offset7, InverseDifferenceMoment_angle135_offset1

InverseDifferenceMoment_angle135_offset4, InverseDifferenceMoment_angle135_offset7

InverseDifferenceMoment_angle45_offset1, InverseDifferenceMoment_angle45_offset4

InverseDifferenceMoment_angle45_offset7, InverseDifferenceMoment_angle90_offset1

InverseDifferenceMoment_angle90_offset4, InverseDifferenceMoment_angle90_offset7

6) Cluster Shade

Formula

$$\sum_{i,j}((i-\mu)+(j-\mu))^{3} g(i,j)$$

Cluster Shade in clustered shading, we group similar view samples according to their position

and, optionally, normal into clusters. In AK Software, we have the 36 parameters related to Cluster analysis, first we describe the 18 related to Cluster Shade.

ClusterShade_AllDirection_offset1, ClusterShade_AllDirection_offset1_SD,

ClusterShade_angle0_offset1, ClusterShade_angle45_offset1,

ClusterShade_angle90_offset1, ClusterShade_angle135_offset1,

ClusterShade_AllDirection_offset4, ClusterShade_AllDirection_offset4_SD,

ClusterShade_angle0_offset4, ClusterShade_angle45_offset4,

ClusterShade_angle90_offset4, ClusterShade_angle135_offset4,

ClusterShade_AllDirection_offset7, ClusterShade_AllDirection_offset7_SD,

ClusterShade_angle0_offset7, ClusterShade_angle45_offset7,

ClusterShade_angle90_offset7, ClusterShade_angle135_offset7

7) Cluster Prominence

Formula

$$\sum_{i,j}((i-\mu)+(j-\mu))^4 g(i,j)$$

Cluster Prominence is a measure of asymmetry of a given distribution, high values of this feature indicate that the symmetry of the image is low, in medical imaging low values of cluster prominence represent a smaller peak for the image grey level value and usually the grey level difference between the forms is small.

ClusterProminence_AllDirection_offset1, ClusterProminence_AllDirection_offset1_SD,

ClusterProminence_angle0_offset1, ClusterProminence_angle45_offset1,

ClusterProminence_angle90_offset1, ClusterProminence_angle135_offset1,

ClusterProminence_AllDirection_offset4, ClusterProminence_AllDirection_offset4_SD, ClusterProminence_angle0_offset4, ClusterProminence_angle45_offset4, ClusterProminence_angle90_offset4, ClusterProminence_angle135_offset4, ClusterProminence_AllDirection_offset7, ClusterProminence_AllDirection_offset7_SD, ClusterProminence_angle0_offset7, ClusterProminence_angle45_offset7, ClusterProminence_angle90_offset7, ClusterProminence_angle135_offset7

8) Haralick Correlation

Formula

$$-\sum_{i,j}\frac{(i,j)g(i,j)-\mu_t^2}{\sigma_t^2}$$

* where μ_t and σ_t are the mean and standard deviation of the row (or column, due to symmetry) sums.

HaralickCorrelation_AllDirection_offset1, HaralickCorrelation_AllDirection_offset1_SD, HaralickCorrelation_angle0_offset1, HaralickCorrelation_angle45_offset1, HaralickCorrelation_angle90_offset1, HaralickCorrelation_angle135_offset1, HaralickCorrelation_AllDirection_offset4, HaralickCorrelation_AllDirection_offset4_SD, HaralickCorrelation_angle0_offset4, HaralickCorrelation_angle45_offset4, HaralickCorrelation_angle90_offset4, HaralickCorrelation_angle135_offset4, HaralickCorrelation_AllDirection_offset7, HaralickCorrelation_AllDirection_offset7_SD, HaralickCorrelation_angle0_offset7, HaralickCorrelation_angle45_offset7, HaralickCorrelation_angle90_offset7, HaralickCorrelation_angle135_offset7

⑵ **Haralick texture features**

Measures the degree of similarity of the gray level of the image in the row or column direction. Represents the local grey level correlation, the greater its value, the greater the correlation;

1) Angular Second Moment

$$f_1 = \sum_{i=1}^{N_g}\sum_{j=1}^{Ng}\left(\frac{P(i,j)}{R}\right)^2 = \sum_i \sum_j p(i,j)^2$$

2) Contrast

The contrast feature is a difference moment of the P matrix and is a measure of the contrast or the amount of local variations present in the image.

$$f_2 = \sum_{k=0}^{N_g-1} k^2 \left\{ \sum_{i=1}^{N_g}\sum_{j=1}^{N_g} \delta_{|i-j|,k}\, p(i,j) \right\} = \sum_{k=0}^{N_g-1} k^2 p_{|x-y|}(k)$$

3) Haralick Entropy

$$f_9 = -\sum_{i=1}^{N_g}\sum_{j=1}^{N_g} p(i,j)\log(p(i,j))$$

4) HaraVariance

$$f_4 = \sum_{i=1}^{N_g}\sum_{j=1}^{N_g} (i-\mu)^2 p(i,j)$$

5) sumAverage

$$f_6 = \sum_{i=2}^{2N_g} i p_{x+y}(i)$$

6) sumVariance

$$f_7 = \sum_{i=2}^{2N_g} (i - f_8)^2 p_{x+y}(i)$$

7) sumEntropy

$$f_8 = -\sum_{i=2}^{2N_g} p_{x+y}(i)\log(p_{x+y}(i))$$

8) differenceVariance

$$f_{10} = \text{variance of } p_{x-y}$$

9) differenceEntropy

$$f_{11} = \sum_{i=0}^{N_g-1} p_{x-y}(i)\log(p_{x-y}(i))$$

10) inverseDifferenceMoment

$$f_5 = \sum_{i=1}^{N_g}\sum_{j=1}^{N_g} \frac{1}{1+(i-j)^2} p(i,j)$$

(3) **Form Factor Parameters**

These group of features includes descriptors of the three-dimensional size and shape of the tumor region. Let in the following definitions V denote the volume and A the surface area of the volume of interest. We determined the following shape and size-based features:

1) Sphericity

$$sphericity = \frac{\pi^{\frac{1}{3}}(6V)^{\frac{2}{3}}}{A}$$

2) Surface area

The surface area is calculated by triangulation (i.e. dividing the surface into connected triangles) and is defined as:

$$A = \sum_{i=1}^{N} \frac{1}{2} |a_i b_i \times a_i c_i|$$

3) Compactness 1:

$$compactness\ \ 1 = \frac{V}{\sqrt{\pi}A^{\frac{2}{3}}}$$

4) Compactness 2:

$$compactness\ \ 2 = 36\pi\frac{V^2}{A^3}$$

5) Maximum 3D diameter:

The maximum three-dimensional tumor diameter is measured as the largest pairwise Euclidean distance, between voxels on the surface of the tumor volume

6) Spherical disproportion:

$$spherical\ \ disproportion = \frac{A}{4\pi R^2}$$

Where R is the radius of a sphere with the same volume as the tumor. Where N is the total number of triangles covering the surface and a, b and c are edge vectors of the triangles.

7) Surfacetovolumeratio:

$$surface\ \ to\ \ volume\ \ ratio = \frac{A}{V}$$

8) VolumeCC

The volume (V) of the tumor is determined by counting the number of pixels in the tumor region and multiplying this value by the voxel size

9) VolumeMM

The maximum 3D diameter, surface area and volume provide information on the size of the lesion. Measures of compactness, spherical disproportion, sphericity and the surface to volume ratio describe how spherical, rounded, or elongated the shape of the tumor is.

(4) **Histogram Parameters**

First-order statistics are concerned with properties of individual pixels.

They describe the distribution of voxel intensities within the CT image through commonly used and basic metrics. Let X denote the three-dimensional image matrix with N voxels and P the first order histogram divided by N_l discrete intensity levels. The following first order statistics were extracted:

1) Energy

$$energy = \sum_{i}^{N} X(i)^2$$

2) Entropy

$$entropy = -\sum_{i=1}^{N_l} P(i) \log_2 P(i)$$

3) Kurtosis

$$kurtosis = \frac{\frac{1}{N}\sum_{i=1}^{N}(X(i) - \bar{X})^4}{\left(\sqrt{\frac{1}{N}\sum_{i=1}^{N}(X(i) - \bar{X})^2}\right)^2}$$

where $\bar{X}$ is the mean of X.

4) MaxIntensity

The maximum intensity value of X.

5) MeanValue

$$mean = \frac{1}{N}\sum_{i}^{N} X(i)$$

6) Mean absolute deviation

The mean of the absolute deviations of all voxel intensities around the mean intensity value.

7) MedianIntensity

The median intensity value of X

8) MinIntensity

The minimum intensity value of X

9) Range:

The range of intensity values of X.

10) Root mean square (RMS):

$$RMS = \sqrt{\frac{\sum_{i}^{N} X(i)^2}{N}}$$

11) Skewness:

Represents the degree of asymmetric distribution in the image histogram. High values of Skewness mean that the distribution is asymmetric otherwise the image is more symmetric; negative skew is when the numerical distribution is relatively long also called negative Skewness distribution, the opposite is referred as positive Skewness distribution (positive skew). It's possible to use the positive and negative Skewness to draw comparisons between the uniform distribution curve.

$$skewness = \frac{\frac{1}{N}\sum_{i=1}^{N}(X(i) - \bar{X})^3}{\left(\sqrt{\frac{1}{N}\sum_{i=1}^{N}(X(i) - \bar{X})^2}\right)^3}$$

where $\bar{X}$ is the mean of X.

12) Standard deviation: stdDeviation

$$standard\ \ deviation = \left(\frac{1}{N-1} \sum_{i=1}^{N} (X(i) - \bar{X})^2 \right)^{1/2}$$

where $\bar{X}$ is the mean of X.

13) Uniformity:

$$uniformity = \sum_{i=1}^{N_l} P(i)^2$$

14) Variance:

$$variance = \frac{1}{N-1} \sum_{i=1}^{N} (X(i) - \bar{X})^2$$

where $\bar{X}$ is the mean of X.

15) VolumeCount

16) VoxelValueSum

Represents the Sum calculations for voxels in the ROI.

17) RelativeDeviation

Let $\bar{X}$ denote the mean of a set of quantities X_i , then the relative deviation is defined by:

$$\frac{\Delta \mathrm{X_i}}{\bar{\mathrm{X}}} = \frac{|\mathrm{X_i} - \bar{\mathrm{X}}|}{\bar{\mathrm{X}}}$$

18) FrequencySize

According to each pixel values in the GLCM.

19) Quantiles

Quantile normalization is a global adjustment method that assumes the statistical distribution of each sample is the same. The normalization is achieved by forcing the observed distributions to be the same and the average distribution, obtained by taking the average of each quantile across

samples. They are cut points dividing the range of a probability distribution into contiguous intervals with equal probabilities or dividing the observations in a sample in the same way.

For a finite population of *N* equally probable values indexed 1, …, *N* from lowest to highest, the *k*-th *q*-quantile of this population can equivalently be computed via the value of:

$$I_p = N\ k/q$$

In AK software, we have 5 Quantiles:

Quantile0.025, Quantile0.25, Quantile0.5, Quantile0.75, Quantile0.975.

20) Percentile

A percentile (or a centile) is a measure used in statistics indicating the value below which a given percentage of observations in a group of observations fall.

The percentile, p%, of a distribution is defined as that value of the brightness *a* such that: P(a) = p%. or equivalently: $\int_{-\infty}^{a} P(\alpha) = p\%$

The P-th percentile $0 < P \leq 100$ of a list of N ordered values (sorted from least to greatest) is the smallest value in the list such that P percent of the data is less than or equal to that value. This is obtained by first calculating the ordinal rank and then taking the value from the ordered list that corresponds to that rank. The ordinal rank *n* is calculated using this formula

$$n = \frac{P}{100} * N$$

AK Software have 19 Percentiles.

Percentile5, Percentile10, Percentile15, Percentile20, Percentile25, Percentile30, Percentile35, Percentile40, Percentile45, Percentile50, Percentile55, Percentile60, Percentile65, Percentile70, Percentile75, Percentile80, Percentile85, Percentile90, Percentile95.

⑸ **Run-length matrices**

The grey level run-length matrix (RLM) P_r (i,j | θ) is defined as the numbers of runs with pixels of gray level i and run length j for a given direction θ. RLMs is generated for each sample image segment having directions (0°,45°,90° &135°), then the following **ten statistical features** were derived: short run emphasis, long run emphasis, grey level non-uniformity, run length non-uniformity, Low Grey Level Run Emphasis, High Grey Level Run Emphasis, Short Run Low Grey Level Emphasis, Short Run High Grey Level Emphasis, Long Run Low Grey Level Emphasis and Long Run High Grey Level Emphasis.

1) Short Run Emphasis (18 Parameters)

Formula

$$SRE(\theta) = \frac{1}{n_r} \sum_{i=1}^{M} \sum_{j=1}^{N} \frac{p(i, j, \theta)}{j^2}$$

ShortRunEmphasis_AllDirection_offset1, ShortRunEmphasis_AllDirection_offset1_SD, ShortRunEmphasis_angle0_offset1, ShortRunEmphasis_angle45_offset1, ShortRunEmphasis_angle90_offset1, ShortRunEmphasis_angle135_offset1, ShortRunEmphasis_AllDirection_offset4, ShortRunEmphasis_AllDirection_offset4_SD, ShortRunEmphasis_angle0_offset4, ShortRunEmphasis_angle45_offset4, ShortRunEmphasis_angle90_offset4, ShortRunEmphasis_angle135_offset4, ShortRunEmphasis_AllDirection_offset7, ShortRunEmphasis_AllDirection_offset7_SD, ShortRunEmphasis_angle0_offset7, ShortRunEmphasis_angle45_offset7, ShortRunEmphasis_angle90_offset7, ShortRunEmphasis_angle135_offset7.

2) Long Run Emphasis (18Parameters)

Formula

$$LRE(\theta) = \frac{1}{n_r} \sum_{i=1}^{M} \sum_{j=1}^{N} p(i, j, \theta)\, j^2$$

3) Grey Level Non-uniformity(18Parameters)

Formula

$$GLN(\theta) = \frac{1}{n_r} \sum_{i=1}^{M} (\sum_{j=1}^{N} p(i, j, \theta))^2$$

4) Run Length Non-uniformity(18Parameters)

Formula

$$RLN(\theta) = \frac{1}{n_r} \sum_{j=i}^{N} (\sum_{i=1}^{M} p(i, j, \theta))^2$$

5) Low Grey Level Run Emphasis(18Parameters)

Formula

$$LGRE(\theta) = \frac{1}{n_r} \sum_{j=i}^{N} \sum_{i=1}^{M} \frac{p(i, j, \theta)}{i^2}$$

6) High Grey Level Run Emphasis(18Parameters)

Formula

$$HGRE(\theta) = \frac{1}{n_r} \sum_{j=i}^{N} \sum_{i=1}^{M} p(i, j, \theta) i^2$$

7) Short Run Low Grey Level Emphasis(18Parameters)

Formula

$$SRLGE(\theta)=\frac{1}{n_r}\sum_{j=i}^{N}\sum_{i=1}^{M}\frac{p(i,j,\theta)}{i^2 j^2}$$

8) **Short Run High Grey Level Emphasis(18Parameters)**

Formula

$$SRHGE(\theta)=\frac{1}{n_r}\sum_{j=i}^{N}\sum_{i=1}^{M}\frac{p(i,j,\theta)i^2}{j^2}$$

9) **Long Run Low Grey Level Emphasis(18Parameters)**

Formula

$$LRLGE(\theta)=\frac{1}{n_r}\sum_{j=i}^{N}\sum_{i=1}^{M}\frac{p(i,j,\theta)j^2}{i^2}$$

10) **Long Run High Grey Level Emphasis(18Parameters)**

Formula

$$LRHGE(\theta)=\frac{1}{n_r}\sum_{j=i}^{N}\sum_{i=1}^{M}p(i,j,\theta)i^2 j^2$$

where n_r is the total number of runs and n_p is the number of pixels in the image.

⑹ **The gray level size zone matrix(GLSZM)**

A GLSZM describes the amount of homogeneous connected areas within the volume, of a certain size and intensity, thereby describing tumor heterogeneity at a regional scale. A voxel is considered connected if the distance is 1 according to the infinity norm. In a GLSZM P(i,j)th element equals the number of zones with gray level I and size j appear in image.

Ng be the number of discreet intensity values in the image

Ns be the number of discreet zone sizes in the image

Np be the number of voxels in the image

Nz be the number of zones in the ROI, which is equal to $\sum^{Ng}_{i=1}\sum^{Ns}_{j=1}P(i,j)$ and $1\le Nz\le Np$

P(i,j) be the size zone matrix

p(i,j) be the normalized size zone matrix, defined as p(i,j)=P(i,j)/Nz

1) **SizeZoneVariability**

Formula

$$SZV = \frac{\sum_{j=1}^{N_s}(\sum_{i=1}^{N_g} P(i,j))^2}{N_z}$$

2) **HighIntensityEmphasis**

Formula

$$HIE = \frac{\sum_{i=1}^{N_g}\sum_{j=1}^{N_s} P(i,j)i^2}{N_z}$$

3) **HighIntensityLargeAreaEmphasis**

Formula

$$HILAE = \frac{\sum_{i=1}^{N_g}\sum_{j=1}^{N_s} P(i,j)i^2 j^2}{N_z}$$

4) **HighIntensitySmallAreaEmphasis**

Formula

$$HISAE = \frac{\sum_{i=1}^{N_g}\sum_{j=1}^{N_s} \frac{P(i,j)i^2}{j^2}}{N_z}$$

5) **IntensityVariability**

Formula

$$IV = \frac{\sum_{i=1}^{N_g} (\sum_{j=1}^{N_s} P(i, j))^2}{N_z}$$

6) **LargeAreaEmphasis**

Formula

$$LAE = \frac{\sum_{i=1}^{N_g} \sum_{j=1}^{N_s} P(i, j)\, j^2}{N_z}$$

7) **LowIntensityEmphasis**

Formula

$$LIE = \frac{\sum_{i=1}^{N_g} \sum_{j=1}^{N_s} \frac{P(i, j)}{i^2}}{N_z}$$

8) **LowIntensityLargeAreaEmphasis**

Formula

$$LILAE = \frac{\sum_{i=1}^{N_g} \sum_{j=1}^{N_s} \frac{P(i, j)\, j^2}{i^2}}{N_z}$$

9) **LowIntensitySmallAreaEmphasis**

Formula

$$LISAE = \frac{\sum_{i=1}^{N_g} \sum_{j=1}^{N_s} \frac{P(i, j)}{i^2 j^2}}{N_z}$$

10) **SmallAreaEmphasis**

Formula

$$SAE = \frac{\sum_{i=1}^{N_g}\sum_{j=1}^{N_s}\frac{P(i,j)}{j^2}}{N_z}$$

11) ZonePercentage

ZP measures the coarseness of the texture by taking the ratio of number of zones and number of voxels in the ROI.

Formula

$$ZP = \frac{N_z}{N_p}$$

4. Detailed descriptions of the statistical methodology

4.1 Decision curve analysis (DCA)

In our study, DCA was used to evaluate the clinical utility of the presented nomogram. DCA assesses prediction models by calculating the range of threshold probabilities in which a prediction or prognostic model was clinically useful. DCA is a compositive method for evaluating and comparing different diagnostic and prognostic models. The theory of DCA can be illustrated by the equation below:

$$\frac{a-c}{d-b} = \frac{1-P_t}{P_t}$$

where d – b represents the influence of unnecessary treatment. If treatment is directed by a prediction model, d – b is the harm related to a false-positive result compared with a true-negative result. Inversely, a – c represents the consequence of rejecting beneficial treatment, in other words, the harm from a false-negative result compared with a true-positive result. Pt represents where the

expected benefit of treatment is equal to the expected benefit of refraining from treatment.

Reference: Vickers AJ, Elkin EB. Decision curve analysis: a novel method for evaluating prediction models. Med Decis Making. 2006 Nov-Dec;26(6):565-74.

4.2 Statistical analysis

The variables were assessed for normal distribution by using the Shapiro-Wilk test. Comparisons of proportions and ranks of variables between training and validation cohorts were performed with the χ^2 or Mann-Whitney U test as appropriate. Median OS and Median TTP was calculated by performing Kaplan-Meier survival analysis, respectively. The potential survival predictors among clinical variables were identified by using a univariate Cox proportional hazards regression analysis approach. The LASSO cox regression algorithm was used with penalty parameter tuning with 10-fold cross-validation, then the multivariate Cox proportional hazards regression model was built. Detailed DCA was provided above.

Statistical analyses were conducted with R software (version 3.5.3, http://www.Rproject.org) and Python3.7. We used the R package "hdnom" to perform the LASSO cox regression analysis and build the multivariate Cox proportional hazards regression model. With these selected predictive features, C-index bootstrapping validation were performed by using the python package "lifeline" to build a Cox regression model. Nomogram construction and calibration curve generation were performed by using the R package "rms". DCA was performed according to its definition using R language.

5. Radiomics score (Rad Score) calculation formula

TTP Rad Score= 0.8456758*ShortRunEmphasis_AllDirection_offset1_SD(art)

-0.6413945*ShortRunHighGreyLevelEmphasis_AllDirection_offset1_SD(art)

-0.4203876*Compactness1(art)

-0.3171338*LowGreyLevelRunEmphasis_AllDirection_offset4_SD(art)

+0.4677059*LargeAreaEmphasis(art)

OS Rad Score= -0.3294247*InverseDifferenceMoment_AllDirection_offset4_SD (art)

-0.4322321*Compactness1(art)

+0.9269416*ClusterShade_AllDirection_offset1(por)

+0.5456392*ZonePercentage(por)

II. Supplementary Tables

Table S2: Univariate Cox proportional hazards regression analysis of clinical parameters for predicting overall survival (OS) and time to progression (TTP) in the training set

	OS		TTP	
Clinical parameter	Hazard Ratio(95% CI)	P value	Hazard Ratio(95% CI)	P value
Sex	1.93(0.58-6.35)	0.28	1.45(0.50-2.51)	0.79
Age (y)	0.96(0.92-1.00)	0.06	0.97(0.95-1.02)	0.36
BMI (kg/m^2)	1.00(0.88-1.14)	1.00	0.98(0.88-1.08)	0.67
Cause of disease	1.92(0.45-8.08)	0.38	1.12(0.43-2.80)	0.85
Child-Pugh class	1.74(0.74-4.10)	0.21	1.39(0.51-2.59)	0.73
ECOG status	1.52(0.76-3.06)	0.24	0.61(0.52-1.67)	0.82
α-fetoprotein level	1.000021（1.000007 -1.000035）	<0.005*	1.00002(1.00001 -1.00003)	<0.005*
Total bilirubin	1.04(1.00-1.09)	0.08	1.02(0.88-1.08)	0.22
Albumin	0.94(0.86-1.03)	0.17	0.98(0.50-2.51)	0.73
Macroscopic vascular invasion	1.30(0.62-2.72)	0.49	1.18(0.43-2.80)	0.82
Extrahepatic spread	0.71(0.34-1.45)	0.34	1.06(0.51-2.59)	0.47
HCC type	0.57(0.28-1.19)	0.13	0.67(0.52-1.67)	0.10
tumor size	1.01(1.00-1.02)	0.09	1.00(1.00-1.02)	0.10

* Indicates a significant difference.

Table S3A: **The features with nonzero coefficients in the least absolute shrinkage and selection operator (LASSO) Cox regression analysis of advanced HCC radiomics features for predicting OS in the training set.**

radiomics features	nonzero coefficients
ClusterProminence_AllDirection_offset1_SD(art)	-0.195
Compactness1 (art)	-0.240
InverseDifferenceMoment_AllDirection_ offset4_ SD(art)	-0.275
LongRunEmphasis_angle135_offset1(art)	0.078
ZonePercentage(art)	0.040
ClusterShade_AllDirection_offset1(por)	0.642
ShortRunEmphasis_AllDirection_offset1_SD(por)	0.136
ZonePercentage(por)	0.320

Note.-HCC = hepatocellular carcinoma, art = late arterial phase, por = portal venous phase.

Table S3B: The features with nonzero coefficients in the least absolute shrinkage and selection operator (LASSO) Cox regression analysis of advanced HCC radiomics features for predicting TTP in the training set.

radiomics features	nonzero coefficients
ClusterShade_AllDirection_offset1(art)	0.150
Variance(art)	-0.110
HaralickCorrelation_AllDirection_offset1 (art)	-0.259
ShortRunEmphasis_AllDirection_offset1_SD(art)	0.656
InverseDifferenceMoment_AllDirection_offset7_SD (art)	-0.045
Compactness1(art)	-0.376
ShortRunHighGreyLevelEmphasis_AllDirection_ offset1_SD(art)	-0.386
LargeAreaEmphasis(art)	0.326
LowGreyLevelRunEmphasis_AllDirection_offset4_SD(art)	-0.373
ZonePercentage(art)	0.073
ClusterShade_AllDirection_offset1D (por)	0.152
GreyLevelNonuniformity_AllDirection_offset1_SD(por)	-0.281

Note.-HCC = hepatocellular carcinoma, art = late arterial phase, por = portal venous phase.

Table S4A: Multivariate Cox proportional hazards regression analyses of advanced HCC radiomics signature for predicting OS in the training set

radiomics signature	Hazard Ratio*	*P*-value
InverseDifferenceMoment_AllDirection_offset4_SD (art)	0.58(0.38-0.90)	0.02†
Compactness1(art)	0.68(0.46-1.02)	0.06
ClusterProminence_AllDirection_offset1_SD(art)	0.55(0.31-0.95)	0.03†
ZonePercentage(por)	1.45(0.98-2.14)	0.06
ClusterShade_AllDirection_offset1(por)	2.62(1.57-4.39)	<0.005†

Note.-HCC = hepatocellular carcinoma, art = late arterial phase, por = portal venous phase.

* Numbers in parentheses are 95% confidence intervals.

† Statistically significant.

Table S4B: Multivariate Cox proportional hazards regression analyses of advanced HCC radiomics signature for predicting TTP in the training set

radiomics signature	Hazard Ratio*	*P*-value
ShortRunEmphasis_AllDirection_offset1_SD(art)	3.30(1.30-8.33)	0.01†
Compactness1 (art)	0.62(0.44-0.87)	0.01†
ShortRunHighGreyLevelEmphasis_AllDirection_offset1_SD (art)	0.46(0.21-1.01)	0.05
LargeAreaEmphasis (art)	1.71(1.19-2.48)	<0.005†
LowGreyLevelRunEmphasis_AllDirection_offset4_SD (art)	0.44(0.24-0.79)	0.01†
HaralickCorrelation_AllDirection_offset1 (art)	0.68(0.36-1.28)	0.24
GreyLevelNonuniformity_AllDirection_offset1_SD (por)	0.55(0.29-1.05)	0.07

Note.-HCC = hepatocellular carcinoma, art = late arterial phase, por = portal venous phase.

* Numbers in parentheses are 95% confidence intervals.

† Statistically significant.

III. Supplementary Figure

Figure S1. Recruitment pathways for patients in this study

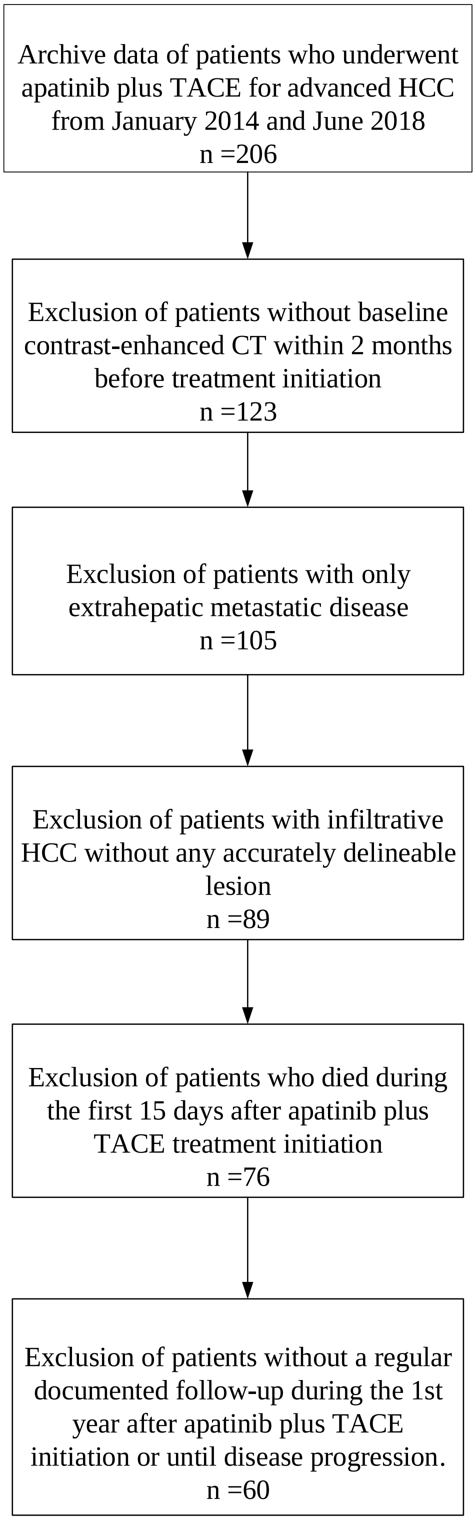

Supplement: Supplementary file 1 — Supplementary figures and tables. [file ijmsv18p2276s1.pdf]
